# Parsers and Grammars: A Tutorial Overview from the Linguistics Building

**DOI:** 10.3390/brainsci12121659

**Published:** 2022-12-03

**Authors:** Carlos Acuña-Fariña

**Affiliations:** Department of English and German, Faculty of Philology, University of Santiago de Compostela, Santiago de Compostela, 15705 Coruña, Spain; carlos.acuna.farina@usc.es

**Keywords:** parsers, grammars, heuristics, strategy, grammatical illusion, template

## Abstract

The purpose of this paper is to re-examine the relationship between grammars and processing systems in light of the various forms of experimental research (especially of an electrophysiological nature) that has been conducted in the last fifteen years or so. First, the notion of ‘processing strategy’ or ‘heuristics processing’ is considered followed by a discussion of structures of great morphosyntactic complexity that parsing systems seem to tackle by simply respecting complex grammatical laws, instead of by resorting to shortcuts. Then, grammatical illusions and what these can teach us about the processing of grammar are considered. It is argued that illusions allow us to discern a few explanatory principles that may redefine the way we see parser–grammar relations. Among these is the idea that how long illusions last in the online-to-offline transition depends in part on their ‘templatability’, that is, the ease with which they become gestaltic templates. Another key idea is that some apparent illusions are in fact nothing more than grammar contemplated at work as in slow motion.

## 1. Introduction

Linguists who take the notion of psychological adequacy seriously are usually not satisfied with what Jackendoff defines as “methodological convenience” [1] (p. 652), which means the inability of grammarians and experimental psychologists to align their scientific agendas at least a little more than they normally do. There is indeed vested interest or convenience in assuming that one’s competence theory of grammar (understood as a formal device that provides a description of the rules/constraints that generate nothing but the infinite set of sentences that are accepted by an ideal native speaker) needs not be backed up in any way by the facts of online performance (understood as the actual use of the linguistic competence taking into consideration time and space limits, see [2]). Seen from the experimental side, methodological convenience also means that any form of moderately sophisticated mentalism (e.g., a grammar ‘rule’) is unrealistic, and that ‘real language’ is something else (most probably just a collection of heuristic strategies). Poeppel and Embick have referred to this trend as “interdisciplinary cross-sterilization” [3] (see also [4] pp. 368–370, [5,6]), and surely many cognitive scientists recognize it well.

One feels that the *Derivational Theory of Complexity* episode has played too big a role in negatively defining the relationships between linguistics and psycholinguistics and, even if we were to endorse the cynical view that the DTC was a complete failure (but see [7]), the failure in question happened over forty years ago. During all those years, and partly as a result of the lessons drawn from it, formal grammars have evolved to become more narrowly focused by lumping a sizable amount of what they had previously purported to cover onto interface areas. Conspicuous among these are constraints on language processing. We are now keenly aware that grammars may be defined in reference to well-formedness conditions but these relate directly to grammaticality judgements which are impacted by limitations of our processing system, especially our short-term memory [8]. It follows that even linguists with no interest in general psychological processes would do well to recognize what a general psychological process is with some accuracy, even if it is only to say, ‘Sorry, I’m not interested, I prefer to stay on this side of the line’. Of course, failure to look out of one’s own box is opportunistic in that it allows linguists of this inclination to proceed at their own pace and, when faced with problems of analysis, to attribute the difficulty to deal with the facts to interface areas that are supposed to be somebody else’s problem. This manner of proceeding guarantees that one cannot fail or loose, but it is patently ad hoc. Naturally, those other linguists who do believe that all of language is a general psychological process would obviously benefit much more from an honest attempt to see what is and what is not ‘core grammar’ vis-à-vis processing, on the hopefully falsifiable assumption that such a concept is viable.

The DTC experience arose largely within the confines of generative grammar and it seems fair to say that minimalism has made great strides in overcoming the inefficiencies that caused it. This probably happened once generative grammarians embraced more realistic theories of competence. Although this is not the place to argue for the merits of current minimalist thinking, it may be added here that this framework now advocates for the role of performance via such notions as interface conditions, spell-out, and the idea of derivation by phases. The idea of computational cost/economy is also relevant, as now, any operation has a cost (for instance Merge is more economical than Move). Memory is also taken into account as derivations are phase bound, not potentially infinite, so access to the results of a previous phase is penalized. With relatively few departures from “orthodox minimalism”, the token transparency idea reappears as a real option [7]. For a (non ‘mainstream’) Merge-based theory that takes into consideration both time and complexity, see [7,9,10,11,12].

It is pertinent to add that some forms of research within the minimalism framework do appear quite similar in spirit to the old idea of the DTC. For instance, Friedmann, as well as Belletti and Rizzi have proposed that the (well-known) greater processing complexity of object relatives vs. subject relatives could be reduced to purely grammatical facts, such as a measurable notion of locality [13,14]. Thus, when a non-local relation is established between A and B and a structural intervener C is in between, “processing difficulty is assumed to be proportional to the number and kind of relevant features shared between the moved item and the intervener” [2].

Another reason why many formal linguists have not made the mental journey back from ‘the DTC failure’ is the fact that that episode was supposed to prove that, unlike time-unconstrained grammatical structure building, online incremental structure building lacks grammatical precision, and therefore, is prone to rest on shortcuts that actually misrepresent the cognitive value of a time-independent grammar. Fortunately, the last forty years of psycholinguistic research have provided a spectacular amount of data on the way the mind and the brain process grammar online. Most of that comes in the form of eye-tracking and ERP studies, with exquisite temporal resolution. If we want to, we are now in a much better position to gauge how precise (or imprecise) grammatical structure building really is, from which we can surely draw valuable conclusions. We are living in the 2020s of the XXI century, not in the 1970s of the previous century.

The purpose of this paper is to discuss the relationship between grammars and processing systems in light of the various forms of experimental research (especially of an electrophysiological nature) that has been conducted in the last 15 years or so. First, in Section 2, going a little further back in time, the notion of ‘processing strategy’ or ‘heuristics processing’ is considered; Section 3 briefly examines the structures of great morphosyntactic complexity that parsing systems seem to tackle by simply respecting complex grammatical laws, instead of by resorting to shortcuts; Section 4 is mostly on illusions, and what these can teach us about the processing of grammar; finally, Section 5 starts from the facts of illusions in the preceding section and suggests a few explanatory ideas that may redefine the way we see parser–grammar relations. Among these ideas is the suggestion that how long illusions last in the online-to-offline transition depends on their ‘templatability’, that is, the ease with which they become gestaltic templates. I believe this is a novel idea that may open up illuminating possibilities for research in the future. Another key idea is that some apparent illusions are, in fact, nothing more than grammar contemplated at work as in slow motion. These are the two main ideas explored, as I believe they hold the best promise there is of finding some new light in this venerable domain of studies. Towards that, I specifically (but necessarily briefly) focus on:

a. Various instances of research aimed at showing the worth of heuristics (e.g., the ‘NVN strategy’, the Extended Projection Principle, garden pathing adverbial clauses, implausible passives, quantifier scope, ambiguous PP attachment, pronominal reference, and ambiguous relative clauses);
b. Various instances of research aimed at showing the worth of blind obedience of grammatical rules (e.g., the LAN component, agreement and unagreement, islands, the filled gap effect, complement clauses with subject gaps, binding relations, and backwards anaphora);
c. Various instances of illusions (e.g., comparatives, negative polarity items, and attraction).

In a way, this contribution is an attempt to gauge the relationship between parsers and grammars from the *token transparency* standpoint [15,16,17], that is, it seeks to argue that modern findings make it possible to significantly reduce the need to separate grammars from processors as much as post-DTC mentalities have suggested we should. In other words, it seeks to argue that alignment between grammars and processors generally prevails and that the idea of a *covering grammar* (completely independent from processing routines) is much less attractive today. This in no way should be taken to mean that there is absolutely no space for heuristic shortcuts in parsing. It means, rather, that if we manage to re-define certain areas of description along new dimensions of analysis, that space becomes much smaller, a fact that—I argue—is significant in and of itself (see for instance [5,12] for similar attempts).

## 2. On Heuristics: Do It Again, or Do It Rough, but Do Not Wait at All!

… and don’t bother to be elegant … Just get the job done.

The cheapest way to start solving the issue of how grammars and processors relate to each other is to assume that there is no ‘relating’ to be done, simply because grammars and processors are one and the same thing (see next section). By the mid1970s, this view was unrealistic because it had just become evident that the formal grammars of the time were not being used in online experiments. Needless to say, one can solve this problem by devising more realistic grammars, but it is useful to remember just what is needed to accomplish that. In the 1970s, it soon became evident that many linguists had not given enough thought to the ordering of elements in a grammatical derivation. Bever, a leading figure in the debates at the time, expresses that accurately in [18] (pp. 387–388):

… the biggest ongoing puzzle presented to psycholinguists concerned with the role of syntax in adult language behavior is the following conundrum:

Sentences are externally serial, (i.e., “horizontal”): derivations are internally hierarchical, (i.e., “vertical”).

That is, the computational domain of a derivation can embrace entire clauses and sentences, while the immediate processing appears to be one word after another.

What this means, among other things, is that a processing system that waits for the right type of information to develop a tidy, sophisticated and perfect grammatical derivation is indeed unrealistic. Processors simply do not have that luxury, and if there is one thing they cannot afford to do, it is wait. It is now well-known that parsing is profoundly *incremental* because we continuously *bet on the horse raced past the barn* [19] (p. 316), [20], that is, we experience garden paths that show that the mind makes ultra rapid structural choices when processing the incoming chain of speech (at a speed of some 15/17 phonemes per second, roughly three/four words). Since left-to-right processing may yield configurations that differ from those developed in the vertical pathway, indeed, a sensible conclusion would be that we do have two systems in place, instead of just one system [8,12,16]. In addition, given the urgency of the job, it also seems logical to assume that the first system to be deployed is the one that is used to deal with the left-to-right needs, not the vertical, time-insensitive grammatically savvy system. Why not give up the second system entirely if the first system is ‘good enough’ [21]? Well, because it may not actually be good enough; symbolic grammars free us from having to deal only with predictable prefab chunks because they capture one of the most important properties of language, i.e., its infinite creativity. There is indeed little (if anything) that symbolic grammar cannot express, and this is crucial because thought seems to be infinite as well. Bever has always made the point that a slow ‘thoughtful’ derivational grammar is also essential to mediate between the needs of production and those of comprehension.

Bever’s work spawned a branch of research sharing the idea that the human mind uses *perceptual strategies* or *heuristics* to quickly get the linguistic job done. These are mental shortcuts that allow humans to solve problems and make judgments swiftly and efficiently and have been described in various ways as: rough-and-ready templates, surface schemata, quick-and-nasty pseudo-grammars, or underspecified but good-enough representations [22,23,24,25,26,27]. They all share a sort of *gestaltic* philosophy and are habitually recognized via three complementary mottos: *late assignment of syntax* (LAST), *semantics proposes and syntax disposes,* and *we understand everything twice*. Although originating in extremely dissimilar theoretical starting points, these processing heuristics are also reminiscent of the notion of *construction*, as in Construction Grammar and its emphasis on simplified symbolic form/meaning packages which result from repeated experience with a particular language [28,29]. 

Bever struck another important key when he made the point that extremely sophisticated grammars such as many of the grammars that populate the linguistics journals are simply unlearnable and that, even if they are used at a second stage of processing, or in parallel with the first, grammars need to be much more surfacey to be realistic. This idea stems from an important theoretical standpoint, i.e., the idea that “how language is produced, comprehended, and learned shapes language structure, and thus must lie at the heart and not the periphery of understanding linguistic structure” [30] (p. 410). If a grammar is the result of usage, it should not be totally unrelated to it. In this type of context, it is often the case that one ends up ignoring the fact that, more often than not, a grammar rule and a heuristic strategy may not actually differ much, or at all (see Section 5). On learnability, this is a key point in the minimalist program agenda [31].

Bever’s ideas also echo part of the philosophy behind currently fashionable cue-based approaches in psycholinguistics [32,33]. It seems fair to say that when very different ideologies converge on some type of rough idea about the nature of language vis-a-vis the nature of processing, the idea in question is likely to have a solid basis in reality. This obviously does not mean that it is all the reality there is. The idea in question emphasizes the ‘sloppiness’ of much language processing and it puts forward ‘a two-stream view’ as opposed to the classic ‘two-stage view’ according to which processing always proceeds from grammar rules first, with encyclopaedic and predictive processes entering the scene as a sort of back-up operation, necessarily during a second stage (as in the classic Garden Path model and its descendants).

Bever’s original heuristics had great intuitive appeal. For instance, the ‘NVN strategy’ captures the idea that a preferred mode of clause construction when facing a DP-VP-DP schema is to assume the semantics of an *action of agent on a patient* type of structure. The canonical ‘word order strategy’ indicates that an DP that appears before a verb phrase will be taken to be a subject in English. In fact, Bever maintained that the *Extended Projection Principle* of generative grammar (i.e., the assumption that clauses must contain subject NPs [34]) was actually based on this strategy that is easy to learn [23] (p. 280). His ‘strategy C’ aimed at plausibility and considered lexical meaning to be responsible for launching sound predictions (predictions are emphasized). In this strategy, a string such as *the lion was killed by the squirrel* would be predicted to take longer to process than controls because the sheer lexicality of the words *lion*, *squirrel,* and *kill* would conjure up the realistic scenario of a lion killing a squirrell, not the other way around. Saffran, Schwartz, and Linebarger showed how aphasic patients struggled with sentences such as *the painting disliked the artist* [35], and ulterior ERP work showed that the brain signature of semantically implausible strings such as *the fox that hunted the poacher* was the P600 wave (roughly, syntactic reanalysis), instead of the N400 (roughly, lexical surprise due to a selection restriction violation, as in *she combed her happiness*), which suggested that by 400 milliseconds after the onset of *the poacher* the parser had not constructed the syntactically determined implausible scenario yet. That is, it suggests that an algorithmic grammar was not consulted first. Note that this interpretation might well be the opposite, i.e., that syntactic structure has already been built, but semantic ’confounds’ force a reanalysis attempt that is captured by the P600, especially given the ‘transitive schema’ that provides a subject DP + a verb + an object DP and the absence of a LAN effect, indicating that syntactic structure violations were not detected (because there are none).

Fernanda Ferreira is usually credited with pushing the heuristics agenda to the present day via her invocation of the idea that syntactic computations and algorithmic procedures are sometimes not only effortful but even completely unnecessary and that, by contrast, heuristics are fast, frugal, and cheap [25,27,36]. From this viewpoint, quite often, linguistic representations are shallow, incomplete, or inaccurate and lacking in detail but still *good enough* to get the job done. Quite often parsing systems simply ignore or do not give due consideration to the actual input and even launch representations that are incompatible with the input if those representations are compatible with the plausibility of events in the real world. Think of the level of analysis that we need to drive a car, for instance. One surely does not need granularity of the type that determines a very precise, indeed millimetric account of distances and shapes (such as the shape of the car that is in front of us); a rough, sloppy understanding of where we are in the landscape and the basic outline of that landscape is usually enough. Perhaps the most shocking example of this type of processing comes from sentences such as Example (1) below:

1. While Anna bathed the baby played in the crib,

In which subjects take *the baby* to be the object of *bath* and the subject of *play* simultaneously, that is they do not fully recover from a garden-path that arises after they first assign the postverbal DP the status of an object phrase and later change that analysis for one in which the DP is a subject of the incoming clause [24]. Another classic case involves implausible passives like *the dog was bitten by the man*, as results show that these are particularly harder to process than controls [21]. Dwivedi studied sentences with quantifier scope ambiguities such as *every kid climbed a tree* [37]. As is well known, these sentences have two possible interpretations: one in which there are several trees (one for each child, the preferred reading) or one with only one tree that all the children climbed. The moving window experiment (self-paced reading) showed that subjects formed a blurred interpretation of the scope possibilities, but question-response accuracy rates indicated that they were less accurate for the non-default reading sentences (one tree for all children), a fact that suggested that algorithmic processing took place *after* the first *dirty* parse. This is also in keeping with research showing that comprehenders may leave pronouns unresolved [38], especially when working memory demands are high or when WM span is small. It has also been suggested that the well-known difficulty of object relatives vs. subject relatives may well be nothing more than a preference for the application of the NVN schema that contains agents that precede patients [39].

Still with relative clauses in mind, Traxler; Pickering, Clifton and Swets; Desmet; and Clifton and Ferreira have shown that ambiguous attachment of relative clauses to complex nominal heads is surprisingly faster than non-ambiguous attachment if participants face only superficial comprehension questions [40,41]. They used sentences such as Examples (2)–(4):

2. The son of the princess who scratched himself in public was terribly humiliated (high attachment of RC to ‘son’);
3. The son of the princess who scratched herself in public was terribly humiliated (low attachment of RC to ‘princess’);
4. The maid of the princess who scratched herself in public was terribly humiliated (globally ambiguous).

When, however, subjects were asked about the ambiguity itself, that advantage largely vanished (though not completely). Finally, in two eye-tracking experiments, Grant and Slogget and Dillon compared ambiguous PP attachment (e.g., *the brother of the waiter with a beard*) and pronominal reference ambiguities (e.g., *we met the brother of the waiter when he visited the restaurant*) in English and found that ambiguities sped, rather than slowed, reading in both cases [42]. It is important not to lose sight of the fact that proponents of good-enough inclinations rarely assume that good-enough strategies are all there is to language processing; rather they tend to highlight the idea that heuristics and syntactic algorithims form a complex interplay [27] (p. 1). This said, the idea of a two-stream system emphasizes parallelism, but nevertheless, it stresses the idea that if a shortcut is available and it gets the job done faster, the sloppy heuristic is expected to be resorted to first.

In this game, one is sure to find disconfirming evidence for almost anything (see for instance [43] for some specific evidence against the *good enough* approach), but on balance, it does seem fair to say that at least a portion of processing is done using strategic shortcuts based on experience and/or encyclopaedic knowledge of the plausibility of events in the world. The question is how much and why.

## 3. Precise Parsing

Despite the comments just made, there is also abundant evidence that parsing can be grammatically very precise, and that precision does not come at the cost of being delayed. For a start, in reference to the three ERP components with the largest psychological evidence across hundreds of experiments over the last three decades, i.e., N400, LAN, and P600, morphosyntactic violations usually begin in the LAN spectrum and end in the P600 family of effects. LAN (left anterior negativity) starts peaking at some 400 ms post anomaly. This is in itself fairly solid evidence that normal syntactic processing is fast and effective, a fact that is easy to forget when one’s research agenda rests on studying the few cases where that may not be observed. Many researchers speak of an eLAN (*Early Left Anterior Negativity* [44]) for clear phrase structure violations (*John’s of picture, instead of *John’s picture of* …), and this operates on the 200–300 ms time window, making morphosyntactic processing potentially even faster. In an overview of some thirty ERP studies of agreement, Molinaro as well as Barber and Carreiras found that the typical malfunction signature for agreement violations in rich inflection languages such as Italian or Spanish (say *la carro or *el barcos) is the biphasic LAN + P600 response (roughly, detection followed by attempts at repair) [45].

So-called *un-agreement* provides a convenient example. The equivalent of appositive phrases such as *we the linguists* or *we linguists* in English can be expressed in Spanish as in Example (5) below, where there is a surface mismatch between the morphological features of the subject phrase (default 3rd person plural) and that of the verb (first person plural). Example (6) offers an instance of standard agreement in that language (note that this is a person feature mismatch, not the habitual number feature mismatch as in English collectives: the committee-sg are-pl gathered).

5. Unagreement:

Los lingüistas (3.pl) escribimos (1.pl) un artículo muy interesante

‘We linguists wrote a very interesting article’

6. Standard agreement:

Los lingüistas (3.pl) escribieron (3.pl) un art culo muy interesante

‘The linguists wrote a very interesting article’

Mancini, Massol, Duñabeitia, and Careiras and Molinaro were able to observe how Spanish parsers ’amnestied’ the construction by suppressing the P600 repair phase, after quickly recognizing the mismatch at the LAN stage [46]. That is, subjects were able to detect the surface mismatch (LAN) of a string that was in any case legal (by 400 ms) and were also able to call off a reaction to it when it became clear that the grammar of Spanish sanctions the mismatch in question. Since the P600 starts peaking at around 600 ms post anomaly and the LAN effect enters the scene at circa 400 ms, this leaves a very narrow time window to operate in. It was already narrow at 400 ms, when the first detection of the legal mismatch was accomplished.

Probably the best illustration of how online processing can display amazing grammatical precision comes from the research on so-called *islands*. As is well-known, in the generative tradition, an island is a construction that does not allow extraction. For instance, unlike in Example (7) below where *who* is supposed to have been extracted from the last position in the structure, after *admired*, in Examples (8) and (9) such moves are not allowed:

7. Who did she hope that the singer said that he admired ---?
8. *Who did the singer read a report that praised ---?
9. *Who did the singer read the Der Spiegel piece about ---?

A very lengthy article in the literature on islands that started with Ross illustrates the complexity of this classic area of the grammar [47] (see also [48,49]). Often, amelioration of the perception of ungrammaticality (a so-called *superiority effect*) is visible when semantic and even pragmatic constraints interfere with the putatively syntactic and construction-specific constraints, making this whole area of grammar really complex [50,51,52]. In this context, it is quite surprising to find out that parsers obey island constraints of an extremely sophisticated nature. The evidence for this comes from failure to obtain *filled gap* (FGE) effects in island domains [53]. This may require a little explaining.

An FGE arises when processors detect a fronted filler such as *who* in Example (10) below, and in order to ease the strain that it causes on short-term memory, they posit a gap at the earliest opportunity. In Example (10) the earliest gap is after *bring* (as in *My brother wanted to know who Ruth will bring* ---. Period), but that position is filled by *us*, so the parser has to overcome its failure and keep on looking for another (which it eventually finds after *to*). We know this happens, because in controls involving no movement such as Example (11), the equivalent to the gap area (the position of *us*) is read faster, which is taken to indicate that in the structures with fillers the unsuccessful attempt to get rid of the filler at that position takes a toll. The FGE:

10. My brother wanted to know **who^i^** Ruth will bring **us** home to **t^i^** at Christmas
11. My brother wanted to know **if** Ruth will bring us home to Mom at Christmas

Stowe suggested that such avid gap filling stops when processors hit islands, and more recent work has refined our knowledge of this fascinating area of research [53]. Studies in English by Sprouse, Wagers and Phillips [54], and Goodal [55], and studies in Spanish by Pañeda, Lago, Vares and Felser [56], among others, have indicated that Stowe was right and also that the suspension of the gap-filling operation was not dependent on individual working memory profiles (memory is needed to keep the displaced filler in a buffer till a gap appears in the left-to-right parse). This confirms that parsers use fine grammatical knowledge online with ease.

In a self-paced reading experiment, Tollan and Palaz further investigated how filler-gap dependencies associated with subject positions were resolved online [57]. They focused on the processing of complement clauses, and found that wh-dependency formation is actively attempted at embedded subject positions (e.g., *Kate in Who did Lucy think Kate could drive us home to*?), unless the embedded clause contains a complementizer (e.g., *Who did Lucy think that Kate* …?). They argue that the absence of the dependency formation in the latter case demonstrates that the parser is sensitive to the so-called *complementizer-trace effect* (e.g., *Who did Lucy think that could drive us home to mom*?), in a manner identical to the suspension of search for fillers in island domains.

The same seems to be evident in studies involving backwards anaphora, and long-distance pronominal dependencies [58]. Consider so-called ‘Principle C’ structures, such as Examples (12)–(14) [34]:

12. *He_i_ likes John_i_.
13. *He_i_ said that John_i_ likes wine.
14. *He_i_ drank beer while John_i_ watched a soccer game

In the generative tradition, Principle C refers to the fact that a pronoun cannot occur in positions where it c-commands its antecedent (though, as a reviewer observes, the fact that pronouns cannot c-command their antecedent is rather more a combination of Principles B and C). Loosely speaking, this means that the pronoun cannot be higher up in the structure than its antecedent, and more often than not this means that it cannot precede it. In three self-paced reading studies, Kazanina, Lau, Lieberman, Yoshida and Phillips studied the way parsers navigated these binding constraints by using manipulations such as Examples (15)–(18) [59]:

15. Principle C/match:

Because last semester she^i^ was taking classes full-time while Kathryn was working two jobs to pay the bills, Erica^i^ felt guilty.

16. Principle C/mismatch:

Because last semester she^i^ was taking classes full-time while Russell was working two jobs to pay the bills, Erica^i^ felt guilty.

17. No constraint/match:

Because last semester while she^i^ was taking classes full-time Kathryn^i^ was working two jobs to pay the bills, Russell never got to see her.

18. No constraint/mismatch:

Because last semester while she^i^ was taking classes full-time Russell was working two jobs to pay the bills, Erica^i^ promised to work part-time in the future.

Note that in the no constraint/mismatch condition (18) we have a tendency to link the pronoun *she* to *Russell*, causing disturbance via elevated reading times (a so-called *Gender Mismatch Effect*). Conversely, in the no constraint/match condition (17) the same operation links *she* to *Kathryn* smoothly (no GME, as *she* and *Kathryn* are gender matched). The contrast between Examples (17) and (18) was not replicated in the pair of Examples (15) and (16) because the parser seems to have realized that the binding of *she* and either *Russell* or *Kathryn* was not legal, so it did not implement it (so there was no GME in Example (16)). In summary, in the no constraint conditions, the urgent need to assign reference to an incoming DP was simply suspended by the online observance of a grammatical rule that disallowed that co-indexation, something equivalent to the c-command requirement. Notice that, as in the case of islands, the structures over which these operations are conducted are quite complex, a fact that might have invited the use of shortcuts. Although we cannot pursue this topic here any further, the reader should know that both online and offline work by Kush, Lidz and Phillips on *Strong* and *Weak Crossover* configurations has also shown that when dealing with the need to bind *wh*-fillers and pronouns, the retrieval of possible distractors is halted when that move forces the parser to ‘disrespect’ the binding constraints (such as c-command and Principle C) at work in these demanding constructions [60].

Long ago, Phillips coined the motto ‘the parser is the grammar’ to refer to this obedient behavior of parsers [7]. If, even when grammar is clearly complex, parsers respect it, what chance is there that they do not when it is easy, as in most other cases? Why not simply adopt the clearly more parsimonious view that parsing involves consulting the grammar only? Or, as Lewis and Phillips put it, why not maintain that “(t)here is no need to specify an additional theory of how the language processor and the grammar interact: if they are the same system, then they do not need to interact” [5] (p. 41).

## 4. It Is Just an Illusion

So it would seem that there are good grounds for assuming that strategies are useful because they are indeed fast, frugal, and cheap at least sometimes (Section 2) and also for assuming that punctilious observance of sophisticated grammatical rules is inescapable many other times (Section 3). This is where a pattern of some sort appears to be lurking and I suggest that the pattern in question may be sensitive to three interacting factors: time, grammatical complexity, and ‘templatability’. I further suggest that the interaction of these three explaining variables is best observed in the behavior of so-called *grammatical illusions*. Let us consider illusions now.

The tem *illusion* brings to mind optical phenomena such as the *Muller-Lyer* illusion, the *Ponzo* illusion, or the *Elephant* illusion and many others where the visual system is tricked into forming representations that actually differ from reality. It is as if the processing system were fooled by deceptive stimuli that make it launch illegal representations. Back to language, consider the famous case of the sentence in Example (19) below [22,61]:

19. *More people have been to Russia than I have.*

Most people experience an illusion of grammaticality (and commonsensicality …) when reading this string and it takes serious reflection to realize that it is meaningless or downright nonsensical, to the point that it ends up becoming impossible to understand (*the number of the people that have been to Russia exceeds the number of me* …???). The semblance of grammaticality seems to stem from the fact that the first clause introduces a comparison between two sets of individuals and the second clause may initially be seen to provide the second set, but it does not (compare, *More math students have been to Russia than Physics students last year*). Townsend and Bever were the first to suggest that the illusion may result from the blending of two constructions [22]: for example, *more people have been to Russia than I* and *People have been to Russia more than I have*. Leivada offers a similar interpretation with *good-enough* undertones [62] (p. 29) (but see [63] for another explanation that results from a recognition of the role of the semantics of the predicate, in particular its *repeatability*).

Upon judging [19] as highly acceptable, naïve participants assign a meaning to it that is related to some aspect of comparison, without necessarily settling on an unambiguous way in either the set comparison or the event comparison interpretation. Under this scenario, participants may not be ready to put their finger on a single, full-fledged, and fully coherent interpretation, but they have parsed the sentence, such that their parser has reached a potentially ambiguous and vague interpretation.

Another lasting illusion arises when reading Example (20):

20. No head injury is too trivial to be ignored.

It is almost impossible not to understand the sentence to mean *No head injury should be ignored—however trivial it may be,* but close inspection reveals that what it really means is *All head injuries should be ignored—including trivial ones* [64]. Consider also a sentence such as *This book fills a much needed gap* [36]. Most people reading it do not even realize that what it actually says is that what is needed is the gap, not the content of the book …(if the gap is needed, one should not fill it …).

As can be seen, just as in the domain of visual illusions, grammatical illusions arise out of a discrepancy between a local parse and a distal one. That is, they arise when the parser starts processing a local string in a particular direction but further processing makes it clear that the initial representation is incompatible with the global interpretation that unfolds later. The typical view of illusions in the literature is that they are a phenomenon that affects processing (real-time structure building), but not so much grammar, since the ungrammaticality becomes evident offline. This may justify a two-system view of the grammar–parser relation [5,12,63]. However, this is not so evident for there seems to be a very obvious gradient in the grammaticality detection threshold, and this can hardly be chance. In short, some illusions are easy to recover from, but others last, often to the point that they become very difficult to undo, even offline, as we have just seen. There must be some reason for this.

In fact, the two most classic and most widely studied illusions involve negative polarity items (NPIs) and agreement *attraction* mistakes, and recent research is showing that the standard assumption that illusions simply disappear offline is too crude even in these classic cases. Take NPIs for a start. Let us, for convenience, adopt the generative description that items such as *ever* or *any* must be c-commanded by a negative element of some sort, as in Example (21) below. Example (22) shows that the c-commanding element (*no*) is necessary:

21. No politician has ever done that.
22. *A politician has ever done that.

Early work by Drenhaus, Saddy and Frisch (speeded grammaticality), Vasishth, Brussow, Lewis and Drenhaus (eye-tracking) on German NPIs showed that (non-c-commanding) illicit licencors fool the processing system in that they confer an appearance of grammaticality to an illegal string [65,66]. For instance, in Example (23):

23. *A politician that no journalist likes will ever say that.

The negative element *no* does not c-command the NPI *ever*, rendering the sentence ungrammatical, but Drenhaus, Saddy and Frisch reported comparatively increased acceptance for the likes of Example (23) than for the likes of Example (22) [65], and Vasishth et al. confirmed the illusion of grammaticality in the RT measures [66]. Vasishth, Brussow, Lewis and Drenhaus reasoned that the processor was fooled because it appeared to have implemented a (rough/dirty/shallow) partial cue-matching check and, since at least one cue (the presence of a previous licensor) is present in Example (23) but not in Example (22), the overall structure was momentarily deemed to be all right (on cue-based processing, see [32,67,68]. Similar work by Xiang, Dillon and Phillips provided confirmatory evidence from the English language [69]. In an ERP study, the authors reported a reduction in the P600 (reprocessing) effect of structures with illicit licensors relative to controls (which, when ungrammatical, resulted in robust P600s). This is known as the *intrusion effect*.

The first indication that NPI illusions are not the monolithic phenomenon that early work seemed to suggest is that, as Parker and Phillips have shown, they can be “switched off by increasing the amount of time from when the potential licensor is processed until the NPI is encountered” [70] (p. 323). Their experiments differ from previous ones in that they focused on the time *before* the NPI became visible, rather than the time available to process it. Example (24) shows how they manipulated time via parenthetical information (underlined):

24. The authors [that no critics recommended for the assignment] have, as the editor mentioned, ever received a pay raise.

Since NPIs display a great deal of cross-linguistic variation (I thank a reviewer for this reminder), it is as well to consider evidence from other languages. In a recent study by Yanilmaz and Drury with Turkish NPIs [71], ERPs showed that subjects fell for the illusion online but, in the offline behavioral results, the intrusion condition was judged with intermediate accuracy (note, ‘intermediate’, not perfect as for grammatical conditions, or clearly degraded as for ungrammatical ones). But the really interesting thing is that similar, but not identical, NPI configurations can fool processors much more, to the point that language-users may be left feeling that perfectly grammatical sentences are not grammatical at all, even offline. According to De Dios, these cases illustrate *illusions of ungrammaticality*. De Dios used structures such as Examples (25)–(27) [72]:

25. The authors [that the critics recommended] have **never** received acknowledgement…
26. The authors [that **no** critics recommended] have **never** received acknowledgement…
27. **No** authors [that the critics recommended] have **never** received acknowledgement…

…for a best-selling novel.

Notice that the three sentences are grammatical but feel very different. Example (25), with single negation, is no doubt comparatively easy to process. Example (26) contains multiple negation, with the negative markers *no* and *never* appearing in different clauses. Example (27) has double negation, but both *no* and *never* appear in the same main clause now. De Dios conducted three experiments (a speeded acceptability task, a self-paced reading task, and an untimed acceptability judgment task) and obtained a cline of results. In the three experiments, the double negation condition, Example (27), exhibited the worst grammaticality judgements and the slowest recovery from disruption. The multiple negation condition, Example (26), exhibited greater acceptability ratings and faster recovery from disruption. Finally, the single negation condition, Example (25), was the fastest to process and judge. As compared with the Xiang et al. study [69], which showed that intrusive *no* improves the perception of *ever* in ungrammatical sentences, De Dios’ experiments prove now that it worsens the perception of never in grammatical ones (this result is hard to accommodate by classic cue-based accounts, since in these, only illusions that rescue ungrammatical segments are deemed possible). Note that the ungrammaticality illusion increased when *no* c-commanded *never*, (27). This condition, in particular, proved to be quite similar to the fake comparison of *More people have been to Russia than I have,* in that subjects had serious trouble recovering from it even in an untimed grammaticality judgement task. This conclusively shows that equating illusions with imprecise and fleeting processing is wrong.

The second most widely studied illusion has to do with agreement mistakes, as in the classic example shown in (28):

28. *The key to the cabinets are getting rusty

Starting in Bock and Miller [73], from which Example (28) is taken, a very large bibliography of so called *attraction* mistakes in production focuses on cases where an illegal attractor (*cabinets*) steals agreement away from the true head of a complex DP (*key*). The last twelve years or so have seen a steady increase in research on attraction from the comprehension standpoint (see [74] and [75] for reviews of production and comprehension research respectively). Descriptively, attraction is seen in comprehension when reading times in the verb area reflect little or no difficulty at all as compared with grammatical controls and when ungrammaticality detection is less accurate [76,77,78]. This *pardoning effect* is often seen by cue-based theoreticians as reflecting a content-addressable architecture where memory chunks of previous regions of the sentence are retrieved in parallel in such a way that the chunk with more matching cues is finally the victor [32,67,68]. In Example (28) *cabinets* and *are* provide partial matching via the shared plural feature. It is fairly obvious that this illusion of grammaticality does not last long, hence, its status in the literature as a classic illusion; it is really easy to recover from it, and therefore, it exemplifies the distance between online and offline behavior (the literature on attraction has recently focused upon whether attraction in comprehension is indeed due to a retrieval process initiated at the verb, so after the DP subject phrase has already been encoded [79], or, instead, to encoding interference, that is difficulty in the initial memory encoding of items (the building of the subject DP node) observable prior to the appearance of a verb [75,80,81,82,83,84]).

This simplified account becomes less simplified when we consider two factors. First, work by Lago, and Acuña-Fariña and Meseguer showed that, similar to Examples (25)–(27) for NPIs, attraction could be observed even in grammatical strings in the shape of increased RTs in the re-reading of the noun and the attractor (before the verb) when these differed in number [75]. However, the attraction effects were much smaller than for ungrammatical strings, something consistent with the fact that they are sometimes not even reported [77,80]. Therefore, this is another cline (more attraction in ungrammatical than in grammatical strings). Second, even the structures most resistant to attraction, reflexives, do show attraction effects in the right circumstances. Reflexives can be manipulated using examples such as Example (30) as compared with Example (29), as Dillon, Mishler, Slogget and Phillips did [81]:

29. The new executive who oversaw the middle manager(s) apparently was | *were dishonest about the company’s profits.
30. The new executive who oversaw the middle manager(s) apparently doubted himself | *themselves on most major decisions.

The authors found that the plural verb *were* in Example (29) showed attraction effects from the plural distractor (*managers*), but that the plural reflexive pronoun *themselves* in Example (30) did not. In the ERP study by Xiang et al. [69], the authors mentioned that reflexives yielded robust P600 effects not affected by the presence of distractors. However, in a new series of eye-tracking experiments, Parker and Phillips proved that even reflexives may elicit intrusion effects when the reflexive and the subject DP mismatch in more than just one cue (animacy + gender, animacy + number, and gender + number) [70]. This is evidence of more gradient behavior, yet again.

## 5. Trying to Link the Dots

Ever since the early work of Miller and Chomsky on the nature of memory (including working memory) in sentence processing [15], it has been known that certain operations that cannot be accomplished quickly and automatically by the grammar or the parser can, nevertheless, be accomplished slowly and deliberately using general cognition. String reversal is a common example; no grammar involves reversing a string, though this is not a computationally complex operation, and humans can slowly and deliberately reverse a string, up to a given length. Therefore, the capacity to reverse a string is not computationally complex, can be done to a limited extent by humans (despite our limited working memory), and does not feature at all in grammar. Thus, these ‘mysteries’ have always perplexed us; the parser does some things very quickly and automatically, but other operations, objectively quite simple, are off-limits altogether. In this vein, and coming back to the issues under consideration here, how can a parser misparse the very simple DP-VP agreement rule in (*the key to the cabinets* + V) but respect extremely complicated island and binding domains? I suggest the answer has to do with a set of interactions involving syntactic complexity, distal vs. proximal processing, and something I can only define as *templatability*, i.e., the inherent potential of a construction to be grasped as a ready-to-use template. I elaborate on this now.

The literature on illusions views these only through the lens of a construction’s *intrusion profile*. However, given that the constructions that have been proven to be susceptible to illusions differ drastically in complexity, their intrusion profile is likely to also depend on the complexity. The constructions tested also differ in the time needed to resolve dependency formation, which can be realized based on, on the one hand, a superficial comparison of DP-VP agreement, and on the other hand, comparisons and negation. Negation and comparisons both involve juggling two sets of entities and their mutual relationships at once (the two terms of the comparison and the negated vs. unnegated facts). This involves conceptual complexity in and of itself. However, agreement is not conceptually challenging for the very good reason that it is not basically a conceptual operation; rather, it is a formal feature-matching operation, i.e., an instruction to link/unify controllers and targets into a phrasal package, independently of their meaning [74,84]. In Spanish, for instance, *las ideas verdes incoloras* (‘colorless green ideas’) is assembled as a DP because the head features of *ideas* (fem + plural) are redundantly replicated on every nominal satellite, but the features themselves (especially the gender feature) tend to be semantically arbitrary (as is the whole DP). Notice also that the sturdiest agreement attraction effects have been obtained for PP modifiers inside a single clause (*the key to the cabinets are rusty), but the single clause is not usually the venue where NPIs and comparisons are tested (see Examples (23)–(26) above). This makes them even harder to deal with. Of course, compound sentences involve a greater need for memory resources to satisfy long-distance dependencies and when, additionally, memory is set to deal with two negated sets of referents against their unnegated baselines, it is surely stretched thin (on the complexities of negation and NPIs, see [85,86,87], among many others. The view that NPIs are licensed in downward entailing and nonveridical contexts is somewhat mainstream, but see Barker for criticism [88]; for a recent overview of negative dependencies, see [89]). A larger and more complicated piece of syntactic structure obviously also makes room for greater time to deploy strategic choices that may be hard to separate from the purely grammatical ones. 

I rush to clarify two things before we proceed any further. First, I am using an intuitive, informal account of complexity and conceptual complexity here. The reader may find more formal, operational accounts of these notions in, for instance, Hawkins ‘s ideas about domain minimization, form minimization, early immediate constituency, and online processing maximation [90,91]. Friedmann et al., interestingly, contemplated the height of the functional layer involved in a minimalist derivation as an index of complexity. Although they meant this in connection with learnability issues, it seems clear that it can apply to other domains of enquiry, such as the present one [13]

Second, one must acknowledge that in accounts such as this, memory is often mentioned too lightly, and this is no exception. As already noted, foundational, theoretical considerations on the relationship between memory and grammar go back to Miller and Chomsky [15], who explored the empirical consequences of the limited nature of human working memory in sentence processing, versus previous idealized models with infinite pushdown stacks, infinite RAM, etc. The fact is that ever since early work by Blaubergs and Braine showed that comprehension was severely compromised in sentences with three or more center embeddings [92], and Wanner and Maratsos suggested that the difficulty of object relatives vs. subject relatives was sensitive to short-term memory [93], research on memory has become a world of its own and requires very careful consideration. Memory load models are now too nuanced and specific, not to be misrepresented when mentioned in passing. Electrophysiologically, early research done with the ERP methodology casts a sustained anterior negativity between fillers and gaps relative to control strings without gaps [94]. This is perhaps the clearest demonstration that memory is the major bottleneck in language processing. Then, in addition to the shape and amount of tree over which a memory unit must be kept in a buffer, elaboration and semantic distinctiveness of the material to store are now known to enhance retrieval. Finally, memory computations are very much affected by competition between the constituents that keep entering the scene incrementally. The cue-based model uses a *content-addressable architecture* to map different forms of interference in retrieval triggered by partial cue-matching and has become impossible not to refer to in current work on any kind of syntactic computation containing long-distance dependencies ([67] and many others). Gibson’s *Dependency Locality Theory* views memory overload as a result of the number of discourse referents introduced along a processing path, and therefore, it is similar to cue-based retrieval (but less adept to deal with NPI configurations or negation, it would appear) [95]. For models that view memory rather as a ‘working space’ defined in reference to the steps needed to complete a derivation while an element is ‘on the stack’, see for instance Kobele, and Gerth and Hale [95], who take classic notions such as the HOLD hypothesis of Wanner and Maratsos and propose a left-to-right minimalist derivational mechanism (based on [96]). Notice that though the level of embedding (as in the sentence *No authors that …*) is a problem for memory, it has long been noted that difficulty (complexity) lies more in the position that specific items take in the structure than in embedding *per se*, the classic case of subject vs. object relatives. See Caplan for a review of models of working memory in sentence comprehension [97].

In addition to *the complexity issue*, *the processing time issue* (whether illusions degrade or even disappear depending on the time mediating the appearance of the potential licensor and the appearance of, say, an NPI) and *the lasting time issue* (whether they linger or not after the dependency is potentially finalized) are also different between attraction and NPIs. The three dimensions differ so much in the two constructions that we are, in fact, entitled to wonder if, rather than a true illusion, attraction is nothing more than an instance of incremental processing where the intruder and the verb are validated locally and fleetingly before distal processing makes it clear that that local validation must be abandoned later. That is, in a string such as *the key to the cabinets are* …, *cabinets are* is not ungrammatical locally (fleetingly), it simply becomes ungrammatical very soon, as soon as the time needed to process the long-distance dependency (subject–verb agreement) is available. This is surely why the illusion of attraction with grammatical materials has proved weak but the illusion of NPIs with grammatical materials is very robust, to judge from De Dios’s recent findings (see Examples (25)–(27) above). That is, we recover from the mini interference caused by a frequent [pl + pl] local match (X X X *cabinets are* X X X) easily but not so from *No authors that the critics recommended have never received acknowledgment for* …. I suggest this is because we fall for a heuristic in the latter but not in the former. In the former, we simply use ‘mini grammar’ locally (local parse of frequent formula: pl + pl), until time for deploying ‘maxi grammar’ becomes available (Lewis and Phillips, 2015). “One representation is favored at one moment and then disfavored just a couple of hundred milliseconds later. But these representations are different steps in the workings of a single parsing mechanism, not the results of separate systems” [9,98,99]. I further suggest that we resort to a heuristic in the case of NPIs because local parsing is impossible and the inherent complexity of the construction makes it useful to ‘template’ it. An island domain is complex but difficult to template, so only algorithmic grammar remains available, but strings involving item-based correlations such as [*No…. ever*] or [*more … than*] are probably worth templating for the very good reason that more often than not the appearance of *ever* is indeed preceded by the appearance of a c-commanding negative element, and the appearance of the first set of a comparison is more often than not accompanied by the appearance of the second set after it (more than a case of if A then B, based on frequency, it may rather be a case of if A, then B is constrained by A. See Chesi for ways of making that kind of implicational thinking more precise in an expectation-based minimalist grammar [99]). The heuristic-driven nature of the processing of these easily templatable domains can also help explain the forming of initial rough and ready but underspecified representations, as usually (but not in the *More people … Russia* … example) these, though sloppy, are still ‘good enough’, given the complexity that must be parsed.

## 6. Conclusions

In summary, first, a brief review of the following has been presented:

a. Various instances of research aimed at showing the worth of heuristics (e.g., the ‘NVN strategy’, the Extended Projection Principle, garden pathing adverbial clauses, implausible passives, quantifier scope, ambiguous PP attachment, pronominal reference, and ambiguous relative clauses);
b. Various instances of research aimed at showing the worth of blind obedience of grammatical rules (e.g., the LAN component, agreement and unagreement, islands, the filled gap effect, complement clauses with subject gaps, binding relations, and backwards anaphora);
c. Various instances of illusions (e.g., comparatives, negative polarity items, and attraction).

Then, it is concluded that if constructions differ in their *templatability* potential, evidence for strategic shortcuts (heuristics) may be expected to correlate more with the easily *templatable* domains, and evidence for dogged observance of complex grammatical constraints may be expected more for less template-driven forms, such as islands. If these ideas are correct, then the classic discussion of whether algorithmic grammar antecedes strategies or strategies antecede algorithmic grammar may revolve around constructing specific profiles. Note that, in a system such as the one that is being sketched here, a (yet-to-be-determined) portion of what heuristics are supposed to cover is covered instead by grammar at different snapshots of time (the transition from mini stretches of grammar to coherent grammatical wholes [9,10,100] and Lewis and Phillip’s “psycholinguistic model of slow linguistic processes” [5]), that is, as mere local vs. distal processing. Additionally, if we recognize that even classic illusions show a cline via their lasting profile, then the distinction between time-sensitive parsing and time-insensitive grammars becomes even more blurred, a fact that contributes to the idea of greater alignment between these two domains.

## Data Availability

Not applicable.

## References

[1] Jackendoff R. (2003). Précis of Foundations of Language: Brain, Meaning, Grammar, Evolution. Behav. Brain Sci..

[2] Chesi C., Moro A. (2015). The subtle dependency between Competence and Performance. MIT Work. Pap. Linguist..

[3] Poeppel D., Embick D., Cutler A. (2005). Defining the relation between linguistics and neuroscience. Twenty-First Century Psycholinguistics: Four Cornerstones.

[4] Ferreira F. (2005). Psycholinguistics, formal grammars, and cognitive science. Linguist. Rev..

[5] Lewis S., Phillips C. (2015). Aligning grammatical theories and language processing models. J. Psycholinguist. Res..

[6] Acuña-Fariña C. (2016). Opportunistic processing of language. Lang. Sci..

[7] Phillips C. (1996). Order and Structure. Ph.D. Thesis.

[8] Phillips C. (2006). The real-time status of island phenomena. Language.

[9] Chesi C. (2015). On directionality of phrase structure building. J. Psycholinguist. Res..

[10] Phillips C. (2003). Linear Order and Constituency. Linguist. Inq..

[11] Phillips C., Lewis S. (2013). Derivational order in syntax: Evidence and architectural consequences. Stud. Linguist..

[12] Momma S., Phillips C. (2018). The relationship between parsing and generation. Annu. Rev. Linguist..

[13] Friedmann N., Belletti A., Rizzi L. (2021). Growing trees: The acquisition of the left periphery. Glossa A J. Gen. Linguist..

[14] Rizzi L. (1990). Relativized Minimality.

[15] Miller G.A., Chomsky N., Luce R.C., Bush R.R., Galanter E. (1963). Finitary models of language users. Handbook of Mathematical Psychology.

[16] Berwick R., Weinberg A.S. (1986). The Grammatical Basis of Linguistic Performance: Language Use and Acquisition.

[17] Stabler E.P. (1984). Berwick and Weinberg on linguistics and computational psychology. Cognition.

[18] Bever T., Sanz M., Laka I., Tanenhaus M. (2013). The Biolinguistics of Language Universals-the next years. Language Down the Garden Path.

[19] Bever T.G., Hayes H. (1970). The cognitive basis for linguistic structure. Cognition and the Development of language.

[20] Sturt P., Lombardo V. (2005). Processing coordinated structures: Incrementality and connectedness. Cogn. Sci..

[21] Ferreira F. (2003). The misinterpretation of noncanonical sentences. Cogn. Psychol..

[22] Townsend D.J., Bever T.G. (2001). Sentence Comprehension: The Integration of Habits and Rules.

[23] Bever T., Piattelli-Palmarini M., Uriagereka J. (2009). Remarks on the Individual Basis for Linguistic Structures. Of Minds and Language: The Basque Country Encounter with Noam Chomsky.

[24] Christianson K., Hollingworth A., Halliwell J.F., Ferreira F. (2001). Thematic roles assigned along the garden path linger. Cogn. Psychol..

[25] Ferreira F., Patson N. (2007). The ‘Good Enough’ Approach to Language Comprehension. Lang. Linguist. Compass..

[26] Slattery T.J., Sturt P., Christianson K., Yoshida M., Ferreira F. (2013). Lingering misinterpretations of garden path sentences arise from competing syntactic representations. J. Mem. Lang..

[27] Karimi H., Ferreira F. (2016). Good-enough linguistic representations and online cognitive equilibrium in language processing. Q. J. Exp. Psychol..

[28] Goldberg A. (1995). Constructions: A Construction Grammar Approach to Argument Structure.

[29] Goldberg A. (2006). Constructions at Work: The Nature of Generalization in Language.

[30] Tanenhaus M., Sanz M., Laka I., Tanenhaus M. (2013). The impact of “The Cognitive Basis for Linguistic Structures”. Language Down the Garden Path.

[31] Chomsky N. UCLA Lectures 2020. https://ling.auf.net/lingbuzz/005485.

[32] Lewis R., Vasishth S., Van Dyke J.A. (2006). Computational principles of working memory in sentence comprehension. Trends Cogn. Sci..

[33] Van Dyke J.A., McElree B. (2006). Retrieval interference in sentence comprehension. J. Mem. Lang..

[34] Chomsky N. (1981). Lectures on Government and Binding: The Pisa Lectures.

[35] Saffran E.M., Schwartz M.F., Linebarger M.C. (1989). Semantic Influences on Thematic Role Assignment: Evidence from Normals and Aphasics. Brain Lang..

[36] Ferreira F., Ferraro V., Bailey K.G.D. (2002). Good-enough representations in language comprehension. Curr. Dir. Psychol. Sci..

[37] Dwivedi V.D. (2013). Interpreting Quantifier Scope Ambiguity: Evidence of Heuristic First, Algorithmic Second Processing. PLoS ONE.

[38] Love J., McKoon G. (2011). Rules of engagement: Incomplete and Complete Pronoun Resolution. J. Exp. Psychol. Learn. Mem. Cogn..

[39] Lin Chien-Jer C., Sanz M., Laka I., Tanenhaus M. (2013). Thematic Templates and the comprehension of relative clauses. Language Down the Garden Path: The Cognitive Basis for Linguistic Structure.

[40] Traxler M., Pickering M., Clifton C. (1998). Adjunct attachment is not a form of lexical ambiguity resolution. J. Mem. Lang..

[41] Swets B., Desmet T., Clifton C., Ferreira F. (2008). Underspecification of syntactic ambiguities: Evidence from self-paced reading. Mem. Cogn..

[42] Grant M., Sloggett S., Dillon B. (2020). Processing ambiguities in attachment and pronominal reference. Glossa A J. Gen. Linguist..

[43] Koring L., Torrens V. (2020). A good-enough representation is not good enough. Typical and Impaired Processing in Morphosyntax.

[44] Friederici A.D., Weissenborn J. (2007). Mapping sentence form onto meaning: The syntax-semantic interface. Brain Res..

[45] Molinaro N., Barber H., Carreiras M. (2011). Grammatical agreement processing in reading: ERP findings and future directions. Cortex.

[46] Mancini S., Massol S., Duñabeitia J.A., Carreiras M., Molinaro N. (2019). Agreement and illusion of disagreement: An ERP study on Basque. Cortex.

[47] Ross J.R. (1967). Constraints on Variables in Syntax. Ph.D. Thesis.

[48] Chomsky N., Anderson S.R., Kiparsky P. (1973). Conditions on transformations. A Festschrift for Morris Hale.

[49] Chomsky N. (1986). Knowledge of Language.

[50] Pesetsky D., Reuland E., ter Meulen A. (1987). Wh-in-Situ: Movement and unselective binding. The Representation of (In)Definitess.

[51] Truswell R. (2011). Events, Phrases, and Questions.

[52] Tanaka M. (2019). Similarities and Differences between Quantifier Raising and Wh Movement Out of Adjuncts. Syntax.

[53] Stowe L. (1986). Models of Gap Location in the Human Language Processor.

[54] Sprouse J., Wagers M., Phillips C. (2012). A test of the relation between working memory capacity and syntactic island effects. Language.

[55] Goodal G. (2016). The D-linking effect on extraction from islands and non-islands. Front. Psychol..

[56] Pañeda C., Lago S., Vares E., Felser C. (2020). Island effects in Spanish comprehension. Glossa A J. Gen. Linguist..

[57] Tollan R., Palaz B. (2021). Subject Gaps Revisited: Complement Clauses and Complementizer-Trace Effects. Front. Psychol..

[58] Van Gompel R.P.G., Liversedge S. (2003). The influence of morphological information on cataphoric pronoun assignment. J. Exp. Psychol. Learn. Mem. Cogn..

[59] Kazanina N., Lau E., Lieberman M., Yoshida M., Phillips C. (2007). The effect of syntactic constraints on the processing of backwards anaphora. J. Mem. Lang..

[60] Kush D., Lidz J., Phillips C. (2017). Looking forwards and backwards: The real-time processing of Strong and Weak Crossover. Glossa A J. Gen. Linguist..

[61] Wellwood A., Pancheva R., Hacquard V., Phillips C. (2018). The anatomy of a comparative illusion. J. Semant..

[62] Leivada E. (2020). Language Processing at Its Trickiest: Grammatical Illusions and Heuristics of Judgment. Languages.

[63] Phillips C., Wagers M., Lau E., Jeffrey R. (2011). Grammatical Illusions and Selective Fallibility in Real-Time Language Comprehension. Experiments at the Interfaces. Syntax & Semantics.

[64] Wason P., Reich S. (1979). A Verbal Illusion. Q. J. Exp. Psychol..

[65] Drenhaus H., Saddy D., Frisch S., Kepser S., Reis M. (2005). Processing negative polarity items: When negation comes through the backdoor. Linguistic Evidence: Empirical, Theoretical, and Computational Perspectives.

[66] Vasishth S., Brüssow S., Lewis R.L., Drenhaus H. (2008). Processing polarity: How the ungrammatical intrudes on the grammatical. Cogn. Sci..

[67] Lewis R., Vasishth S. (2005). An activation-based model of sentence processing as skilled memory retrieval. Cogn. Sci..

[68] Vasishth S., Lewis R.L. (2006). Argument-head distance and processing complexity: Explaining both locality and anti-locality effects. Language.

[69] Xiang M., Dillon B., Phillips C. (2009). Illusory licensing effects across dependency types: ERP evidence. Brain Lang..

[70] Parker D., Phillips C. (2016). Negative polarity illusions and the format of hierarchical encodings in memory. Cognition.

[71] Yanilmaz A., Drury J.E. (2018). Prospective NPi licensing and intrusion in Turkish. Lang. Cogn. Neurosci..

[72] De Dios-Flores I. (2019). Processing sentences with multiple negations: Grammatical structures that are perceived as unacceptable. Front. Psychol..

[73] Bock K., Miller C.A. (1991). Broken agreement. Cogn. Psychol..

[74] Acuña-Fariña J.C. (2012). Agreement, attraction and architectural opportunism. J. Linguist..

[75] Lago S., Acuña-Fariña C., Meseguer E. (2021). The reading signatures of agreement attraction. Open Mind..

[76] Pearlmutter N.J., Garnsey S.M., Bock K. (1999). Agreement processes in sentence comprehension. J. Mem. Lang..

[77] Wagers M., Lau E., Phillips C. (2009). Agreement attraction in comprehension: Representations and processes. J. Mem. Lang..

[78] Acuña-Fariña C., Carreiras M., Meseguer E. (2014). Gender and Number Agreement in Comprehension in Spanish. Lingua.

[79] Vasishth S., Nicenboim B., Engelmann F., Burchert F. (2019). Computational models of retrieval processes in sentence processing. Trends Cogn. Sci..

[80] Lago S., Shalom D.E., Sigman M., Lau E.F., Phillips C. (2015). Agreement attraction in Spanish comprehension. J. Mem. Lang..

[81] Dillon B., Mishler A., Slogget S., Phillips C. (2013). Contrasting intrusion profiles for agreement and anaphora: Experimental and modeling evidence. J. Mem. Lang..

[82] Villata S., Franck J. (2020). Similarity-based interference in agreement comprehension and production: Evidence from object agreement. J. Exp. Psychol. Learn. Mem. Cogn..

[83] Smith G., Franck J., Tabor W. (2021). Encoding interference effects support self-organized sentence processing. Cogn. Psychol..

[84] Acuña-Fariña C. (2018). Aspects of a psychologically informed theory of agreement. Folia Linguist..

[85] Baker C.L. (1970). Double Negatives. Linguist. Inq..

[86] Ladusaw W.A., Lappin S. (1996). Negation and Polarity Items. The Handbook of Contemporary Semantic Theory.

[87] Giannakidou A., von-Heusinger K., Maienborn C., Portner P. (2011). Negative and positive polarity items. Semantics: An International Handbook of Natural Language Meaning.

[88] Barker C. (2018). Negative polarity as scope marking. Linguist. Philos..

[89] Giannakidou A.A., Zeijlstra H., Everaert M., van Riemsdijk H. (2017). The Landscape of Negative Dependencies: Negative Concord, N-Words, Split Scope. The Blackwell Companion to Syntax.

[90] Hawkins J. (1994). A Performance Theory of Order and Constituency.

[91] Hawkins J. (2004). Efficiency and Complexity in Grammars.

[92] Blaubergs M.S., Braine M.D. (1974). Short-term memory limitations on decoding self-embedded sentences. J. Exp. Psychol..

[93] Wanner E., Maratsos M., Halle M., Bresnan J., Miller G.A. (1978). An ATN approach to comprehension. Linguistic Theory and Psychological Reality.

[94] Phillips C., Kazanina N., Abada S.H. (2005). ERP effects of the processing of syntactic long-distance dependencies. Cogn. Brain Res..

[95] Kobele G.M., Gerth S., Hale J.T., Morrill G., Nederhof M.-J. (2013). Memory resource allocation in top-down minimalist parsing. Lecture Notes in Computer Science.

[96] Rambow O., Joshi A.K., Clifton C., Frazier L., Rayner K. (1994). A processing model for free word order languages. Perspectives on Sentence Processing.

[97] Caplan D., Hickock G., Small S.M. (2016). Working memory and sentence comprehension. Neurobiology of Language.

[98] Chesi C. (2021). Expectation-Based Minimalist Grammars. arXiv.

[99] Chesi C., Canal P. (2019). Person Features and Lexical Restrictions in Italian Clefts. Front. Psychol..

[100] Gibson E., Karlgren H. (1990). A computational theory of processing overload and garden-path effects. Proceedings of the 13th Conference on Computational Linguistics.

